# Functional Connectivity Underlying Symptoms in Preschool Boys With Autism: A Resting-State Functional Magnetic Resonance Imaging Study

**DOI:** 10.3389/fnins.2022.844821

**Published:** 2022-04-14

**Authors:** Zhihong Lan, Shoujun Xu, Xiangrong Yu, Zhenjie Yu, Meng Li, Feng Chen, Yu Liu, Tianyue Wang, Yunfan Wu, Yungen Gan, Guihua Jiang

**Affiliations:** ^1^Department of Medical Imaging, Zhuhai People’s Hospital, Zhuhai Hospital Affiliated With Jinan University, Zhuhai, China; ^2^Department of Radiology, Shenzhen Children’s Hospital, Shenzhen, China; ^3^Department of Medical Imaging, Guangdong Second Provincial General Hospital, Guangzhou, China

**Keywords:** autism spectrum disorders, functional connectivity, resting-state fMRI, preschool boys, visual

## Abstract

**Background:**

Single-sex children have been regarded as one of the best subjects to understand the abnormal development patterns of autism spectrum disorders (ASDs). However, the functional connectivity (FC) behind their symptoms is still unknown.

**Methods:**

Based on FC analysis, the acquired resting-state functional magnetic resonance imaging (rs-fMRI) data sets, including 86 boys with ASD and 54 normal controls (NC), were used to detect the neural synchronous activity between brain regions. Pearson correlation analysis was used to evaluate the relationship between the abnormal FC value and clinical features.

**Results:**

Individuals with ASD showed enhanced FC between the right calcarine and the right lingual gyrus (LG). The right medial orbital frontal cortex also showed increased FC with bilateral inferior temporal gyrus (ITG) [two-tailed, voxel-level *p* < 0.001, gaussian random field (GRF) correction, cluster-level *p* < 0.05]. We did not find a correlation between the abnormal FC value and clinical scales.

**Conclusion:**

Our study reveals a possible relationship between atypical visual attention and poor learning ability in subjects with ASD, and delayed social language development may be a secondary symptom to ASD.

## Introduction

Autism spectrum disorder (ASD), which includes two core symptoms, persistent defects in social communication and social interaction, and restricted, repetitive patterns of behavior, interests, or activities, is a heterogeneous neurodevelopmental disorder with many individuals affected. Recently, the first national survey of ASD in China has shown that the prevalence of ASD in children aged 6–12 years has been as high as 0.7% (Zhang and Han, 2020), of whom more than 40% receive more than one psychotropic drug treatment due to various symptoms (Henneberry et al., 2021). However, owing to the heterogeneity and high comorbidity rate of ASD (Leader et al., 2021), the typical developmental disorder model of ASD has not been explored so far, which poses great challenges to the treatment and prognosis of ASD. Thus, it remains a focus to explore the typical abnormal development pattern of ASD.

Existing studies have found that in early childhood, the brain volume of individuals with ASD is larger than that of normal children (Hazlett et al., 2017). However, owing to the stagnation or deterioration of cognitive and behavioral functions in late childhood, adolescence, or early adulthood, the brain volume of individuals with ASD seem to be no different to that of normal controls (NCs), known as “pseudo normalization” (Zielinski et al., 2014). Thus, the brain changes associated with “normalization” of brain volume in autism are likely inherently pathological or a complex mixture of pathology, compensatory mechanisms, relatively normal processes, and silent sculpting of the brain by the often atypical life experiences of individuals with autism. Moreover, an increasing number of studies have confirmed that sex differences in ASD are not only reflected in the incidence rate which is two to five times more common in men than in women, but also on the basis of clinical manifestations and anatomy (Lai and Szatmari, 2020). This means that using single-sex subjects in early childhood is one of the most promising ways to explore a more pure, uncompensated model of typical ASD developmental disorders. However, despite the fact that ASD can be early diagnosed by 24 months, the average age of diagnosis is 2 years later (Roberts et al., 2019), so preschool age may be the appropriate age to study ASD.

In our previous study on preschool boys with ASD using regional homogeneity analysis, we found that the right medial orbital frontal cortex (mOFC, which explains the ASD’s repetitive stereotyped behavior), the opercular part of the left inferior frontal gyrus (IFG operc), the left middle temporal gyrus (MTG), and the left angular gyrus (AG, which can explain social language development defect) had abnormal spontaneous brain activity, as well as the right calcarine, a part of the primary visual cortex (Lan et al., 2021). Repetitive and rigid behaviors and social language development disorders are undoubtedly the most obvious clinical symptoms in ASD, and the strengthening of visual ability also seems to be important in the early stage of ASD. For example, an experiment demonstrated that if a toddler spent ≥69% looking at geometric scenes, the positive predictive effectiveness of accurately classifying children with ASD was 100% (Venker et al., 2021). Moreover, the enhanced visual ability of children at high-risk of ASD at 9 months was related to their severity of ASD symptoms at 15 and 24 months (Varcin and Jeste, 2017). Taken together, these findings suggest a link between increased visual ability and ASD symptoms.

We sought to investigate how the discovered brain regions could explain the important symptoms of ASD synergies with other brain regions? Functional connectivity (FC) of resting-state functional magnetic resonance imaging (rs-fMRI), an analytical method for identifying resting state neural networks between brain regions and elucidating abnormal brain activity from a comprehensive perspective, provides a good means of observation (Lowe et al., 1998). Chen et al. (2018) first explored atypical brain FC in preschool individuals with ASD, and found that brain regions involved in social cognition with ASD showed under-connectivity, while those involved in sensory and visual movement showed over-connectivity. McKinnon et al. (2019) found that the restricted behavior was associated with more positive FC between the default mode and dorsal attention networks at 24 months in individuals with ASD. However, some of these studies did not control the sex factor. Furthermore, most of the previous studies on FC in children with ASD investigated the integral network characteristics, but ignored the details of the possible effects of specific brain regions.

To understand the neural mechanism of male ASD symptoms, we attempted to explore the specific association between abnormal spontaneous activity brain regions and other certain brain regions in preschool boys with ASD using a relatively large sample size. Based on the results of a previous regional homogeneity of rs-fMRI study, we defined the right mOFC, left IFG operc, left MTG, left AG, and right calcarine as the region of interest (ROI) to analyze the FC between each of them and the whole brain. Then, correlation analysis was performed with the FC value and ASD scales. Based on a literature review, we speculated that there would be over-connectivity between visual-related brain regions and under-connectivity between the regions associated with social language in preschool-age boys with ASD.

## Materials and Methods

### Participants

Eighty-six preschool boys (3–6 years old) with ASD were recruited from Shenzhen Children’s Hospital, and 54 age-matched boys with typical development were recruited from local advertisements as the NC group. None of the NCs had reported a history of serious medical problems or neurological/mental illness. All participants were native Chinese speakers, their guardians fully understood the purpose of the study and written informed consent was obtained before enrolling in the group. The study was approved by the ethics review committee of Shenzhen Children’s hospital.

Subjects with ASD were only enrolled after being jointly diagnosed by two or more deputy chief physicians of pediatrics or psychiatry. All individuals with ASD met the DSM-5 and were scored for symptom severity using the Autism Behavior Checklist (ABC) and the Childhood Autism Rating Scale (CARS). The CARS and ABC are the main diagnostic and screening tools for children with ASD in China. Although the diagnostic sensitivity of CARS for ASD is not as high as that of the Autism Diagnostic Observation Schedule (ADOS), which is considered as the gold standard, it has higher specificity and can avoid over diagnosed preschool children from being included in the ASD group (Randall et al., 2018). The CARS, which was scored by professionally trained pediatricians and psychiatrists, contains 15 items, and each item is divided into 4 levels. A total score <30 is classified as non-ASD, a total score of 30–36 is classified as mild to moderate ASD, and a total score >36 is classified as severe autism. The ABC, which was completed by guardians (usually parents) who have spent a long time with the children, contains 57 items, each of which is divided into 4 levels. A total score of >30 indicates the suspected presence of ASD symptoms, and a total score of ≥67 indicates the presence of ASD symptoms. We also used the development diagnosis scale for children aged 0–6 years to evaluate the children’s development quotient (DQ) (DQ < 70 is a low score). The NC group did not receive the corresponding scale score. Children with known mental, neurological (such as epilepsy and Tourette’s syndrome), or genetic (such as fragile X and Rett syndrome) disorders were excluded from the ASD group. Children who had a history of unconsciousness for more than 5 min and were currently taking psychoactive drugs were also excluded.

### Imaging Acquisition

Resting-state functional magnetic resonance imaging data were collected in the Radiology Department of Shenzhen Children’s hospital using a 3.0T Siemens Skyra scanner. The Rs-fMRI acquisition parameters were as follows: repetition time/echo time, 2 s/30 ms; slice thickness, 3 mm with a 0.72 mm gap; field of view, 230 mm × 230 mm; flip angle, 90°; and matrix, 64 × 64. Thirty-five axonal slices covering the whole brain were positioned along the AC–PC line, and 240 volumes were acquired over approximately 8 min. Meanwhile, a T1-weighted sequence of magnetization-prepared rapid acquisition gradient echo (MPRAGE) prepared by three-dimensional magnetization covering the whole brain (176 sagittal sections) was obtained. Whole-brain T2-weighted images and T2-FLAIR images were also obtained to rule out the presence of organic brain lesions. After the MRI scan, the images of each participant were examined to ensure that the images met the experimental requirements.

Each child was sedated with 50 mg/kg chloral hydrate by trained and certified nurses in accordance with the guidelines and protocols developed by the hospital Radiation Sedation Committee. During the scan, a foam pad was used to prevent head movement, and adhesive earmuffs was used to protect hearing. Additionally, each participant was required to have a caregiver and their guardian present during the scan.

### Functional Magnetic Resonance Imaging Data Pre-processing

Image pre-processing was performed using the data processing assistant in the resting state fMRI toolbox (DPARSF 3.0 Advanced Edition).^1^ First, we eliminated the data with head movement >1.5 mm or >1.5° in any direction to minimize the influence of head movement. We also removed the first 10 time points of each subject to avoid the signal change before the magnetic field reached the stable state and let the subjects get accustomed to the noise of the fMRI. Next, the T1 image for each subject was co-registered with the functional images of the same subject. This co-registered T1 image was then segmented and normalized to the standard Montreal Neurological Institute (MNI) space (age 4.5–8.5 years) (Fonov et al., 2011) and the voxel size was resampled to 3 mm × 3 mm × 3 mm. After normalization, a full-width Gaussian kernel of 8 mm × 8 mm × 8 mm was used to smooth the image at the half-maximum value, and then the image was processed by linear detrending. Twenty-seven covariates were used for regression analysis, including 24 head motion parameters, white matter signal, global mean signal, and cerebrospinal fluid signal. Finally, bandpass filtering (0.01–0.08 Hz) was applied.

### Functional Connectivity Analysis

The brain regions with abnormal spontaneous brain activity in previous studies of preschool boys with ASD (Lan et al., 2021) were defined as the ROI, with a radius of 6 mm. These five brain regions were the right calcarine (9, −90, 3), right mOFC (6, 51, −9), left MTG (−42, −48, 9), left AG (−42, −57, 27), and left IFG operc (−39, 3, 21). FC analysis was used to explore the patterns between the seed ROI and the whole-brain voxels. For each subject, FC correlation plots of each ROI were obtained by voxel multiple regression, and the resulting correlation coefficients were converted into Z-scores by Fisher’s transformation.

### Statistics

The two-sample *t*-test was used to assess age differences between the ASD and NC groups. The two-sample *t*-test was also executed by the toolbox in DPARSF to identify significant between-group differences in the FC of each ROI. Then, gaussian random field (GRF) was used for multiple correction to determine the brain regions with statistical differences (two-tailed, voxel-level *p* < 0.001, cluster-level *p* < 0.05). Finally, SPSS 20.0 software was used to conduct the Pearson correlation analysis between the FC value of abnormal brain region and the CARS and ABC (*p* < 0.05).

## Results

### Demographic and Clinical Characteristics

As is shown in Table 1, there were no significant between-group differences in age (*T* = −1.518, *p* = 0.13). The values of the ABC, CARS, and DQ of the ASD group were consistent with the ASD standard.

**TABLE 1:** Demographic and clinical characteristics of boys with ASD and NCs.

	ASD group (*n* = 86)	NC group (*n* = 54)	T	*P*
Age	3.92 ± 0.95	4.09 ± 0.96	−1.518	0.13
ABC	68.12 ± 15.15			
CARS	34.17 ± 2.08			
DQ	53.44 ± 7.90			

*Data are presented as the mean ± standard deviation.*

*ABC, autism behavior checklist; CARS, childhood autism rating scale; DQ, developmental quotient.*

### Alterations of Functional Connectivity in Preschool Boys With Autism Spectrum Disorder

**FIGURE 1:**
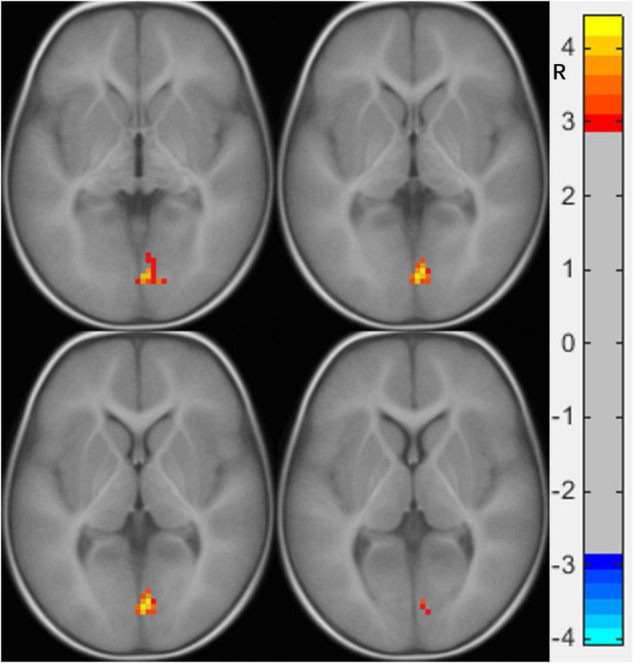
Functional connectivity differences of the right calcarine between preschool boys with ASD and NCs. The warm color (right lingual gyrus) represents increased connectivity.

**FIGURE 2:**
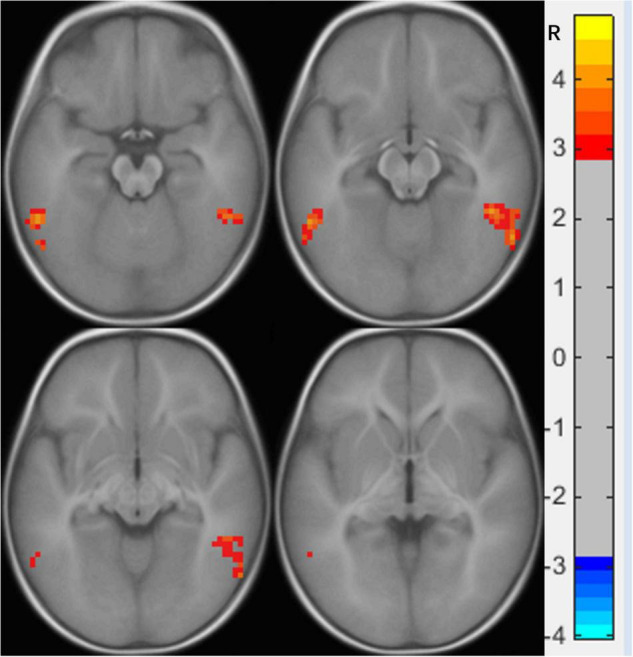
Functional connectivity differences in the right medial orbital frontal cortex between preschool boys with ASD and NCs. The warm color (bilateral inferior temporal gyrus) represents increased connectivity.

Compared to the NC group, the right calcarine-based FC analysis revealed increased FC with the right lingual gyrus (LG) (Figure 1 and Table 2), the right mOFC-based FC analysis revealed increased FC with bilateral inferior temporal gyrus (ITG) (Figure 2 and Table 3), and left MTG-, the left AG-, and the left IFG operc-based FC analyses did not find any significant difference in the brain region.

**TABLE 2:** Brain regions showing abnormal FC with the right calcarine in the ASD group (*p* < 0.05).

Brain area (AAL)	VOXEL	MNI			Peak
		x	y	z	
Lingual_R	102	3	−81	0	4.4347

*R, right; MNI, Montreal Neurological Institute; FC, functional connectivity.*

**TABLE 3:** Brain regions showing abnormal FC with the right medial orbital frontal cortex in the ASD group (*p* < 0.05).

Brain area (AAL)	VOXEL	MNI			Peak
		x	y	z	
Temporal_Inf_L	96	−57	−51	−15	4.9136
Temporal_Inf_R	201	54	−48	−21	4.5960

*L, left; R, right; MNI, Montreal Neurological Institute; FC, functional connectivity.*

### Correlation Between Functional Connectivity Values and Clinical Scales

The FC values of the three brain regions and the values of ABC and CARS had a normal distribution, so Pearson correlation analysis was used. However, we found no significant correlation between any brain region and ASD related scales.

## Discussion

In this study, we used the right calcarine-, right mOFC-, left IFG operc-, left MTG-, and left AG-based FC to compare the differences in the connectivity patterns between preschool boys with ASD and the NC group. We found that the FC between the right calcarine and the right LG, and the FC between the right mOFC and the bilateral ITG were increased in the ASD group. However, no abnormal brain regions were found in FC analysis based on ROI brain regions that are associated with social language. Moreover, no significant correlation was found between the abnormal brain regions and the ASD scale score.

We found that the FC of preschool boys with ASD was increased between the right calcarine and the right LG, both of which are important components of the visual system. The right calcarine belongs to the V1 area, which is responsible for projecting the received visual signals to higher-level processing areas, while the right LG belongs to zone V4, which is more inclined to process detailed color information (Johnson et al., 2015). The FC between the two was increased, which indicates that compared to the NC, the visual system of preschool boys with ASD showed abnormal enhancement in the processing of color information. A previous study found that children with ASD completed the semantic task of picture-word matching more accurately than word-word matching (Turnbull et al., 2020). Another study found that compared to NC children, children with ASD (3 years old) showed faster response time and higher accuracy in completing the task of finding specific graphics hidden in colorful pictures (Nilsson Jobs et al., 2018). The advantage found in these studies in children with ASD in processing color-related details is consistent without our results showing FC enhancement in the right LG and right calcarine. Thus, our findings provide some evidence for visual advantage in children with ASD (Stevenson et al., 2018) from the perspective of functional imaging.

An FC increase between the right mOFC and the bilateral ITG of preschool boys with ASD was also found in our study. The right mOFC is part of the reward loop and is related to the ASD symptoms of restricted, repetitive patterns of behavior, and interests, which includes atypical visual attention (Carlisi et al., 2017), while the bilateral ITG is responsible for the key area of visual association learning (Zhang et al., 2016). The enhanced FC between the two suggests that most of the knowledge obtained by preschool boys with ASD from external visual information is limited to atypical visual attention, which is difficult to separate and transfer due to dysfunction of the reward loop. A previous study found that children with ASD prefer to focus on significant visual objects (such as color and geometric patterns) than NC children (spend more time looking at social stimuli), and the more severe the symptoms, the worse the learning ability of children with ASD (Venker et al., 2021). Another study also found that children with ASD who spend more time watching geometric images will show more serious ASD symptoms, a lower intelligence quotient, and lower adaptive skills (Bacon et al., 2020). These findings support our observed results that enhanced FC between the right mOFC and the bilateral ITG hinders the development of learning ability from the external visual environment in children with ASD. This also explains why children with ASD have visual advantages over NC, but their clinical symptoms show various social disorders. We speculate that the FC enhancement between the right mOFC and the bilateral ITG may be the key to the symptoms of ASD.

Additionally, in contrast to other FC studies of older subjects with ASDs (Chouinard et al., 2017; Kana et al., 2017; Lee et al., 2020), although the brain regions related to social language showed decrease spontaneous brain activity in our previous study, we did not find any other brain region with abnormal FC with them. We speculate that the previously observed abnormal FC in brain regions associated with social language might be functional compensation developed in children with ASD. In most cases, the behavioral symptoms of ASD did not completely appear before the age of two (Zwaigenbaum et al., 2015). However, atypical visual attention, which was not regarded as the core symptom, was manifested in individuals with ASD since infancy (Wang et al., 2015). This precursor symptom of atypical visual attention will interfere with word learning and lead to a delay in social language development in children with ASD (Venker et al., 2018). Therefore, we boldly speculated that the social language development disorder, which was the core symptom most easily observed by parents, may be a secondary result of the abnormal FC between the brain regions related atypical visual attention and visual association learning.

Our research has some limitations. First, our subjects were sedated in the scanner that the results we observed may not fully reflect the changes of FC in the awake state of ASDs. Second, our study was focused on male individuals with ASD and could not be extended to female individuals with ASD. To avoid the over-diagnosed preschool boys being included in the ASD group, the CARS and ABC were selected, but the weight of atypical visual attention was relatively low in these scales, and may mask the correlation between our observed results and the severity of ASD symptoms. Additionally, owing to the sample size, our research focused only on the group-level differences, thus limiting our ability to identify meaningful ASD subgroups, which may help us understand the convergence and divergence mechanism in the brain of preschool boys with ASD.

The current study found that preschool boys with ASD have a certain visual advantage, and that the increased FC between the right mOFC and the bilateral ITG, which caused a reduction in learning ability because of atypical visual attention, may be the key to the symptoms of ASD. Additionally, the social language development delay, which was a significant clinical symptom of ASD, may be a secondary result of atypical visual attention. Indeed, early defects in the field of visual behavior are beneficial to the early diagnosis of ASD and assist with the planning of corresponding treatment.

## Data Availability Statement

The raw data supporting the conclusions of this article will be made available by the authors, without undue reservation.

## Ethics Statement

The studies involving human participants were reviewed and approved by the Ethics Review Committee of Shenzhen Children’s hospital. Written informed consent to participate in this study was provided by the participants’ legal guardian/next of kin.

## Author Contributions

ZL, ZY, ML, FC, YL, and TW draft the manuscripts. SX, XY, YW, and GJ fund the project. YG scan the subjects. All authors contributed to the article and approved the submitted version.

## Conflict of Interest

The authors declare that the research was conducted in the absence of any commercial or financial relationships that could be construed as a potential conflict of interest.

## Publisher’s Note

All claims expressed in this article are solely those of the authors and do not necessarily represent those of their affiliated organizations, or those of the publisher, the editors and the reviewers. Any product that may be evaluated in this article, or claim that may be made by its manufacturer, is not guaranteed or endorsed by the publisher.

## Footnotes

1
http://rfmri.org/DPARSF

## Abbreviations

FC: functional connectivity
ASD: autism spectrum disorder
NC: normal controls
GRF: gaussian random field
mOFC: medial orbital frontal cortex
IFG operc: inferior frontal gyrus
MTG: middle temporal gyrus
AG: angular gyrus
rs-fMRI: resting-state functional magnetic resonance imaging
ROI: region of interest
ABC: autism behavior checklist
CARS: childhood autism rating scale
ADOS: autism diagnostic observation schedule
DQ: development quotient
MPRAGE: magnetization-prepared rapid acquisition gradient echo
MNI: Montreal Neurological Institute.
